# C_60_Br_24_/SWCNT: A Highly Sensitive Medium to Detect H_2_S via Inhomogeneous Carrier Doping

**DOI:** 10.1021/acsami.1c16807

**Published:** 2021-12-06

**Authors:** Jin Zhou, Mohammad Bagheri, Topias Järvinen, Cora Pravda Bartus, Akos Kukovecz, Hannu-Pekka Komsa, Krisztian Kordas

**Affiliations:** † Country Microelectronics Research Unit, Faculty of Information Technology and Electrical Engineering, University of Oulu, P.O. Box 4500, FIN-90014 Oulu, Finland; ‡ Interdisciplinary Excellence Centre, Department of Applied and Environmental Chemistry, University of Szeged, Rerrich Bélatér 1, H-6720 Szeged, Hungary

**Keywords:** H_2_S gas sensor, ppb, brominated fullerene, C_60_Br_24_/SWCNT composite, carrier doping

## Abstract

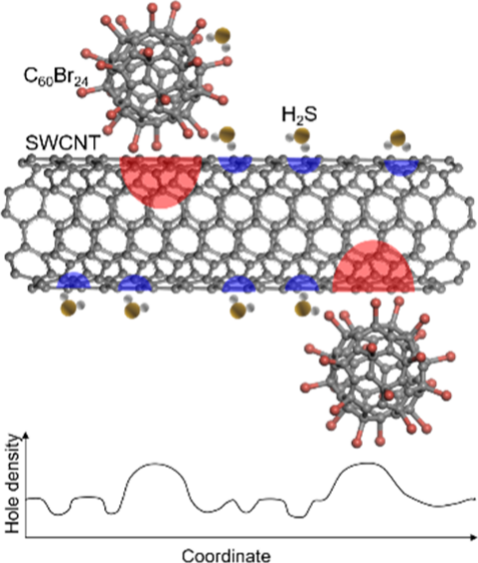

H_2_S is a toxic and corrosive gas, whose accurate detection at sub-ppm concentrations is of high practical importance in environmental, industrial, and health safety applications. Herein, we propose a chemiresistive sensor device that applies a composite of single-walled carbon nanotubes (SWCNTs) and brominated fullerene (C_60_Br_24_) as a sensing component, which is capable of detecting 50 ppb H_2_S even at room temperature with an excellent response of 1.75% in a selective manner. In contrast, a poor gas response of pristine C_60_-based composites was found in control measurements. The experimental results are complemented by density functional theory calculations showing that C_60_Br_24_ in contact with SWCNTs induces localized hole doping in the nanotubes, which is increased further when H_2_S adsorbs on C_60_Br_24_ but decreases in the regions, where direct adsorption of H_2_S on the nanotubes takes place due to electron doping from the analyte. Accordingly, the heterogeneous chemical environment in the composite results in spatial fluctuations of hole density upon gas adsorption, hence influencing carrier transport and thus giving rise to chemiresistive sensing.

## 1 Introduction

Hydrogen sulfide (H_2_S) is a corrosive, toxic, and colorless gas, which accompanies several industrial and natural processes, and may pose health risks upon extended exposure even at sub-ppm concentrations. The acceptable ambient concentration of H_2_S for humans shall be below 100 ppb.^1^ On the other hand, as a biomarker, the detection of H_2_S has also been applied in the early diagnosis of lung diseases.^2,3^ Therefore, sensors that can detect H_2_S rapidly and selectively at the ppb level are of great significance. In recent years, H_2_S sensors with different detection modalities have been developed, including chemiresistive,^4,5^ electrochemical,^6,7^ and surface acoustic wave devices.^8−10^ Among these, chemiresistive sensors have attracted great attention due to their remarkable advantages including low cost, simple fabrication and operation, high response, and easy integration with electronics.^11−13^

A typical chemiresistive sensor is composed of electrodes (either coplanar or interdigitated) defined on a substrate and a sensing material layer that bridges the gap between them. The resistance of chemiresistive gas sensors changes in the presence of gases and the magnitude of change is related to the gas concentration as well as to the strength of gas–surface interactions. The change of resistance is a consequence of (partial) electron transfer or polarization in the sensing material induced by the adsorbed molecules. Typically, the effect on the conductivity is high when the interaction is strong, for example, upon the adsorption of molecules with highly reducing or oxidizing properties, which may facilitate strong dipole attraction or bonds with ionic or covalent character.^14−17^ The drawback of such strong interactions is the poor reversibility, that is, after gas exposure, the analyte cannot desorb quickly, thus compromising sensor recovery. Conversely, weak interactions (e.g., by dispersion forces) generally accompany only small charge transfer between the sensing material and the analyte, which results in minute changes in the carrier concentration and thus a small sensory signal, although often with good desorption and sensor recovery.^18−20^ Hence, it is a plausible strategy to find a medium interaction strength between analytes and sensing materials to ensure optimal sensing performance of chemiresistive gas sensors. While several studies have dealt with optimizing the interaction between analytes and sensing materials,^14−16^ halogen bonding has not received the attention it deserves, despite having similar characters to hydrogen bonding exploited widely for gas sensing due to its moderate strength.^17−20^

In the last few decades, a variety of materials including semiconducting metal oxides, transition metal sulfides, carbon nanomaterials, conducting polymers and organic materials, and their composites were explored for the detection of H_2_S gas at low concentrations.^21−25^ While semiconducting metal oxide-based sensors show good sensitivity, their typical major limitations are high operating temperatures and poor selectivity. In the case of organic semiconducting sensors, their poor conductivity, which originates from the charge-hopping mechanism, limits the overall performance.^26^ Therefore, current efforts aim at combining several different functional materials together to simultaneously address challenges related to good electrical conductivity and gas-sensing capability at room temperature.^27−31^

In this work, we report on nanocomposites of brominated fullerene (C_60_Br_24_) and single-walled carbon nanotubes (SWCNTs) as promising sensing media, which were immobilized on interdigital electrodes of chips using a simple brush-coating method to obtain chemiresistive sensor devices. The sensor exhibits a high response, good repeatability, and selectivity for H_2_S with a measured detection limit of a 25 ppb concentration. Density functional theory (DFT) calculations indicate that adsorption of H_2_S on C_60_Br_24_ has a medium bond strength and it increases the hole density in CNTs near the contact. On the other hand, direct adsorption of H_2_S on SWCNTs causes electron doping and thus results in a spatial fluctuation of the carrier concentration along the nanotubes giving rise to local barriers and scattering centers that limit carrier transport therein. Accordingly, our study shows a feasible approach to sensitize CNTs with halogenated carbon nanomaterials having ideal properties for gas-sensing applications.

## 2 Results and Discussion

### 2.1 Materials Properties

**Figure 1:**
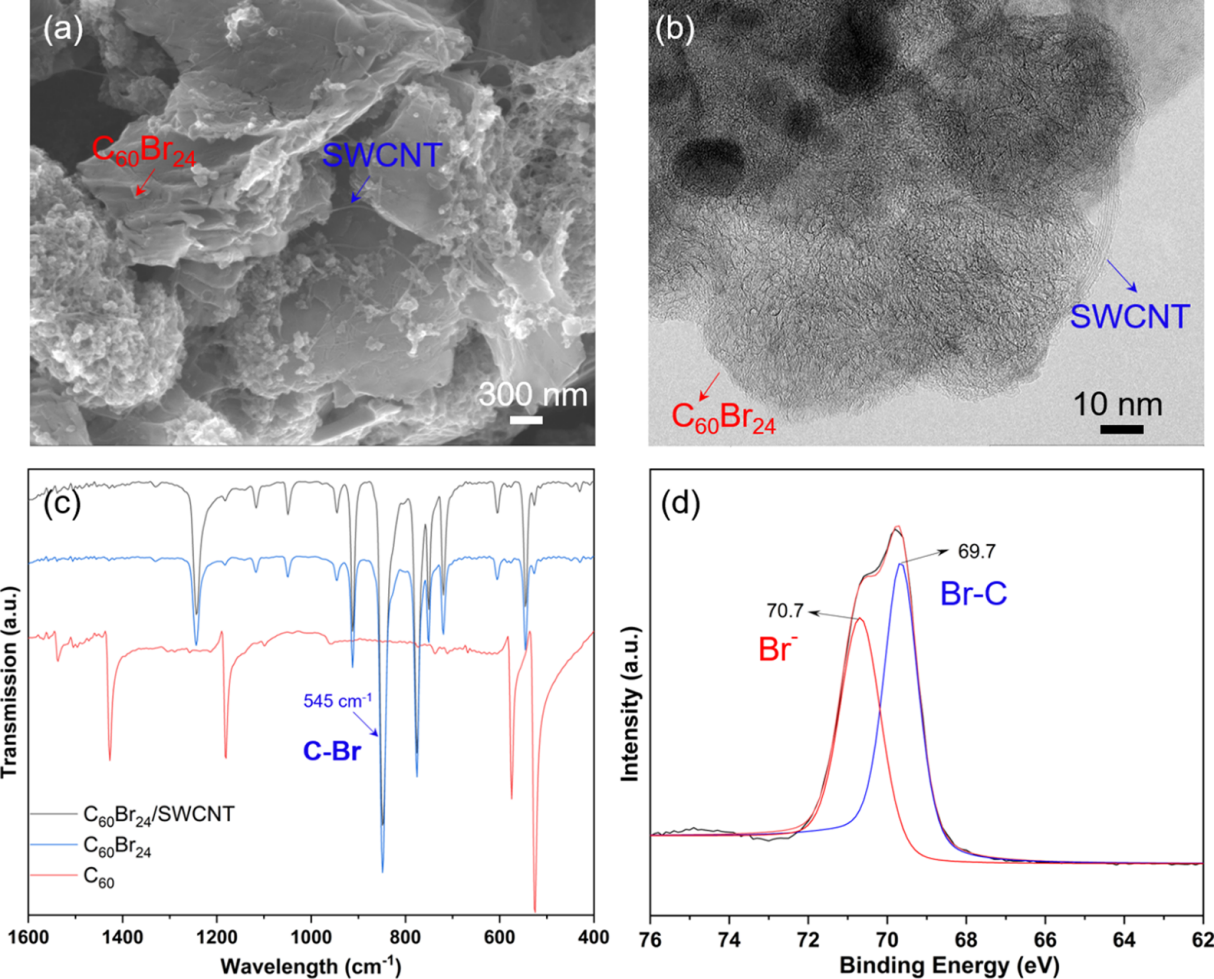
(a) SEM and (b) TEM images of C_60_Br_24_; (c) FTIR spectra of C_60_Br_24_, C_60_, and the C_60_Br_24_/SWCNT composite; and (d) resolved XPS Br 3d peak of C_60_Br_24_.

C_60_Br_24_ was synthesized by the reaction of fullerene (C_60_) with the Br_2_ liquid in the presence of a FeBr_3_ catalyst at room temperature as described earlier by Djordjević and co-workers.^32^ Composites of C_60_Br_24_/SWCNTs were prepared by blending C_60_Br_24_ with SWCNTs in different mass ratios. Scanning and transmission electron microscopy images (Figure 1a,b) show good uniformity of fullerenes on the nanotubes. Peaks in the Fourier-transform infrared (FTIR) spectrum of C_60_Br_24_ (Figure 1c) are nearly identical to those reported earlier.^33^ Peaks at 603 and 545 cm^–1^ originate from radial motions of carbon skeletons partially localized on the functionalized fragments of C_60_Br_24_. The peak at 848 cm^–1^ is due to C=C–Br–C and C=C–Br deformations. The peaks at 1118 and 1224 cm^–1^ indicate the C–C–Br and C–C stretching vibrations, respectively.^34−36^ In addition, compounding C_60_Br_24_ with SWCNTs does not seem to change the chemical structure according to the FTIR spectrum of C_60_Br_24_ in the C_60_Br_24_/SWCNT composite. To get further insights into the chemistry of the brominated fullerenes, X-ray photoelectron spectroscopy (XPS) was carried out. The Br 3d peak (Figure 1d) can be deconvoluted into two peaks corresponding to two components with energies typical for covalent C–Br bonds.^37^ The Raman spectrum of C_60_Br_24_ is very similar to that of C_60_ (Figure S1a). The X-ray diffraction pattern of C_60_Br_24_ (Figure S1b) is consistent with the literature^32^ and shows a series of additional reflections compared to pristine C_60_ indicating that packing of the crystals is different after bromination. The presence of side groups leading to a less dense packing^38^ is supported by the lower intensity and a shift of the main reflections to lower angles, when comparing the X-ray diffraction (XRD) pattern of C_60_Br_24_ to that of pristine C_60_. Thermogravimetric analysis (TGA) of C_60_Br_24_ indicates its high thermal stability with an onset of 5% weight loss at 151 °C (Figure S1c).

### 2.2 Gas-Sensing Behavior

**Figure 2:**
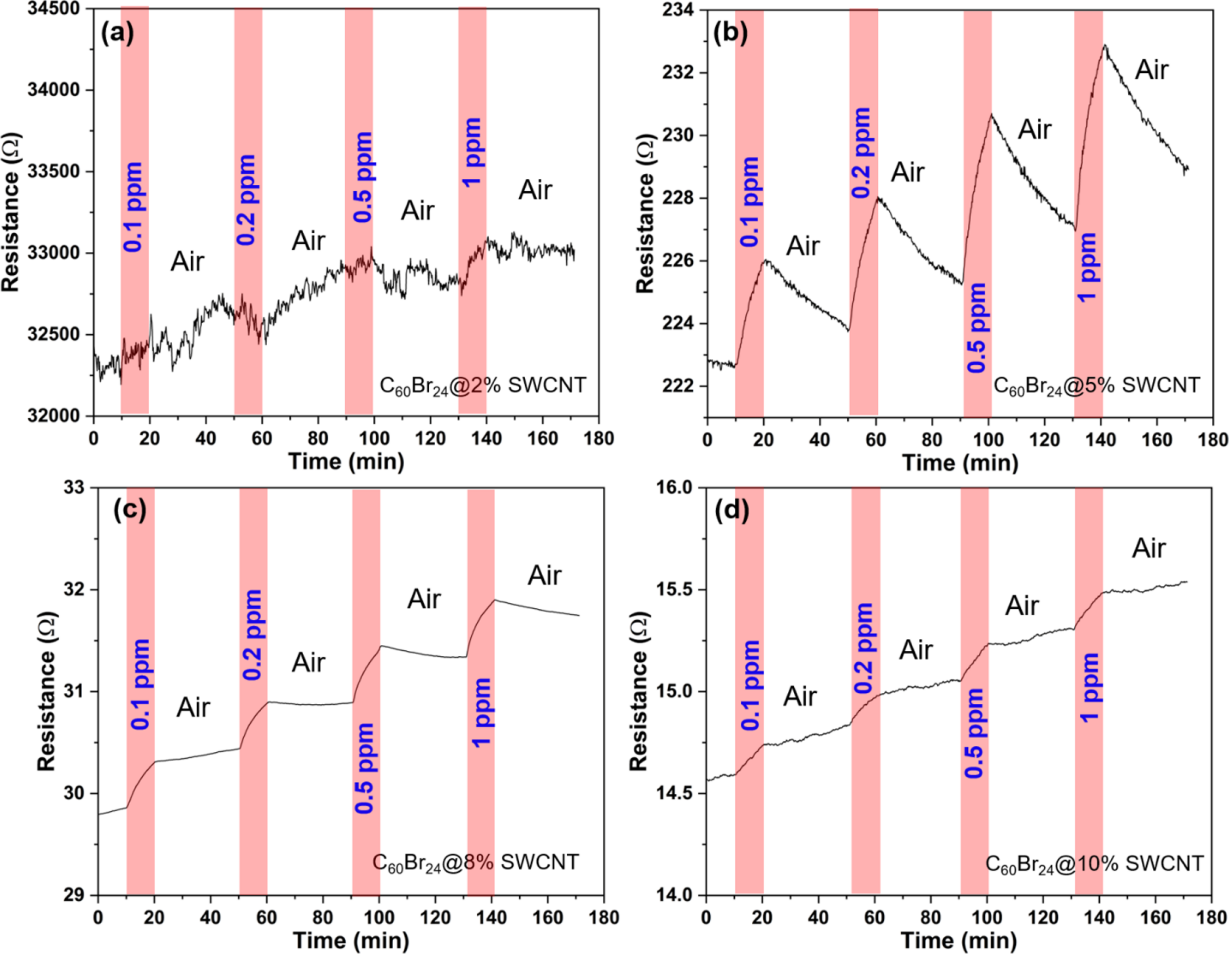
Real-time response–recovery curves of sensors based on C_60_Br_24_/SWCNT composites having different SWCNT loadings: (a) 2, (b) 5, (c) 8, and (d) 10 wt %.

C_60_Br_24_/SWCNT composites having different nanotube loadings (from 2 to 10 wt %) were prepared and then deposited onto ceramic chips having printed Ag–Pd interdigital electrodes to find the optimum composition for H_2_S detection (Figure 2). The high conductance and the fairly linear *I*–*V* characteristics of the fabricated sensors indicate good percolation of the metallic SWCNTs in the network and suggest ohmic contact between the sensing film and the electrodes (Figure S2). At SWCNT concentrations of 5% or higher, the resistance of the sensor devices is well below 1 kΩ and the noise of the real-time response curves is visibly low in contrast to the composite with a 2% SWCNT content. Although the composites with 8 and 10 wt % nanotube contents have a lower resistance than that with a 5 wt % composite, the sensor response of those devices is clearly inferior, which is due to the highly percolated metallic SWCNTs in the network, which practically shunt the semiconducting elements and junctions (that eventually play the main role in sensing with SWCNTs).^39^ As expected, in the extreme condition of having only SWCNTs in the sensor, the response (displayed in Figure S3) is rather poor, in contrast to the best performing C_60_Br_24_/SWCNT composites with a 5 wt % SWCNT loading, which has been selected for further analysis in this work.

**Figure 3:**
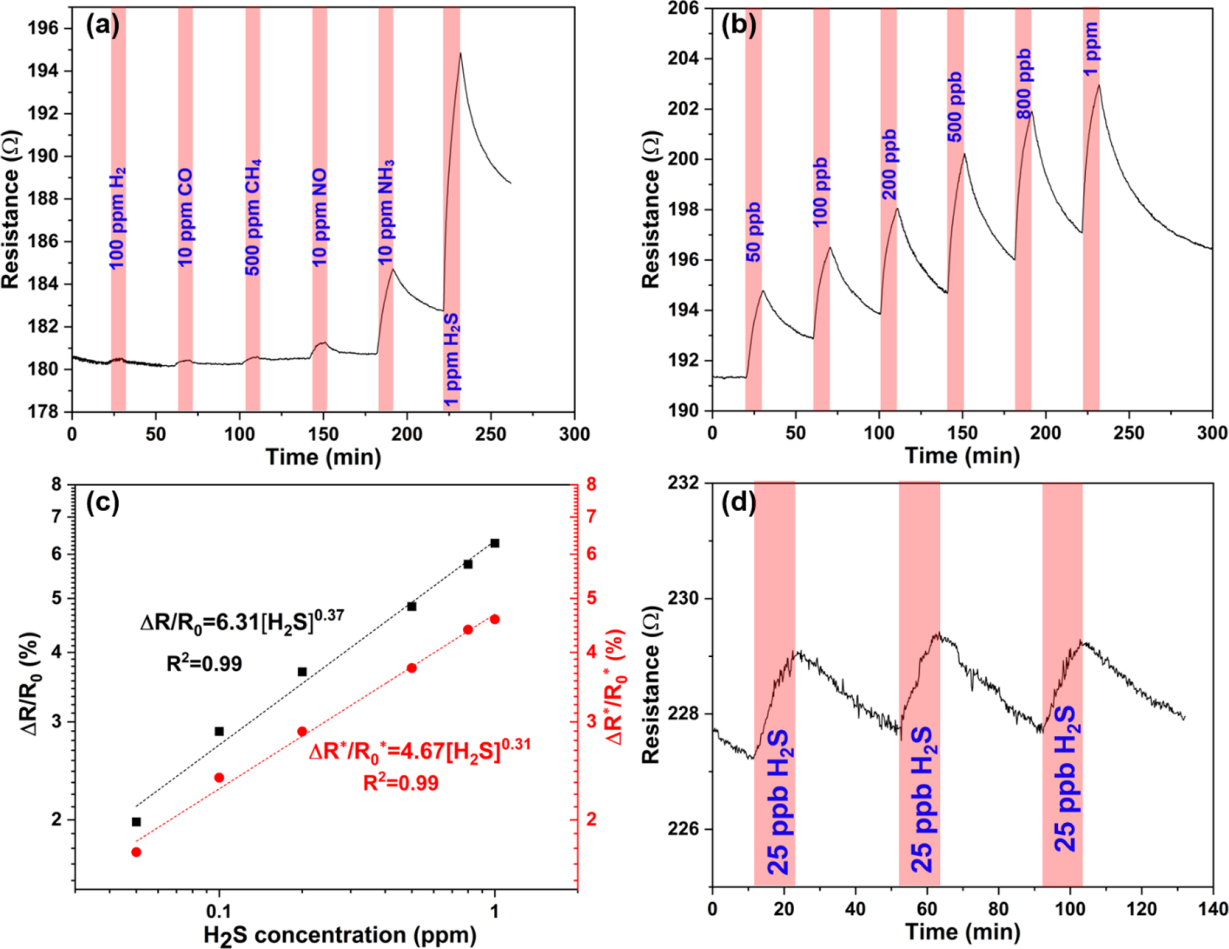
(a) Gas response of the C_60_Br_24_/SWCNT composite (5 wt % SWCNTs) to various analytes in air buffer. (b) Transient response curves measured for H_2_S at concentrations from 50 ppb to 1 ppm. (c) Gas response vs H_2_S concentration (black) and calibration curves (red) of the C_60_Br_24_/SWCNT composite (5 wt % SWCNTs). (d) Real-time device resistance for repeated pulses of 25 ppb H_2_S.

First, to evaluate the selectivity of the sensor, the response in six different gases including H_2_, CO, CH_4_, NO, NH_3_, and H_2_S was measured. As shown in Figure 3a, H_2_S induces the highest increase of resistance among all gases even though its concentration was the lowest, which indicates very good selectivity to this analyte. Due to the insignificant response of sensors to H_2_ and CH_4_ (even at high gas concentrations), we selected CO, NO, and NH_3_ as the main interference gas to further explore the cross-selectivity of the sensor in gas mixtures (Figure S4). The results show that the presence of CO or NO does not influence the sensor response to H_2_S. Contrarily, when a pulse of 10 ppm NH_3_ is introduced to the measurement chamber, the response of the sensor to it is superposed on the original response to H_2_S, which indicates that the composite cannot distinguish these two gases from each other. Gas concentration-dependent real-time resistance curves from 50 ppb to 1 ppm are plotted in Figure 3b, from which we calculate the responses of the sensor with and without considering the baseline drift as shown in Figure S5. By plotting both sets of response data as a function of H_2_S concentrations, we obtain the sensor calibration curves, which follow power functions with fractional exponents similar to those typical for heterogeneous adsorption according to the Freundlich model (Figure 3c). The sensor exhibited a good response of 1.75% at as low as 50 ppb H_2_S. It is important to note that the true response of the sensors is actually higher than the values we calculated because the gas pulse durations (10 min) we applied are not long enough to reach sensor saturation. Experiments with extended pulses of 1 h and an even longer duration as shown in Figures S6 and S4, respectively, indicate that the saturation requires ∼1 h and the response ∼2–3 times higher than that measured after 10 min. On the other hand, the extended gas pulse measurements result in a limited recovery (and maybe partial poisoning) of the sensors because the measured response for subsequent gas pulses with gradually increased H_2_S concentrations is decreased (Figure S6).

Repeatability of sensor data is a critical key performance indicator for applications. As shown in Figures 3d and S7, repeated gas pulses produce nearly identical responses. It is also worth mentioning that based on the noise and the eventual response of the devices, the estimated detection limit of H_2_S with the C_60_Br_24_/SWCNT composite (5 wt %) is 1 ppb (Figure S8).

**Figure 4:**
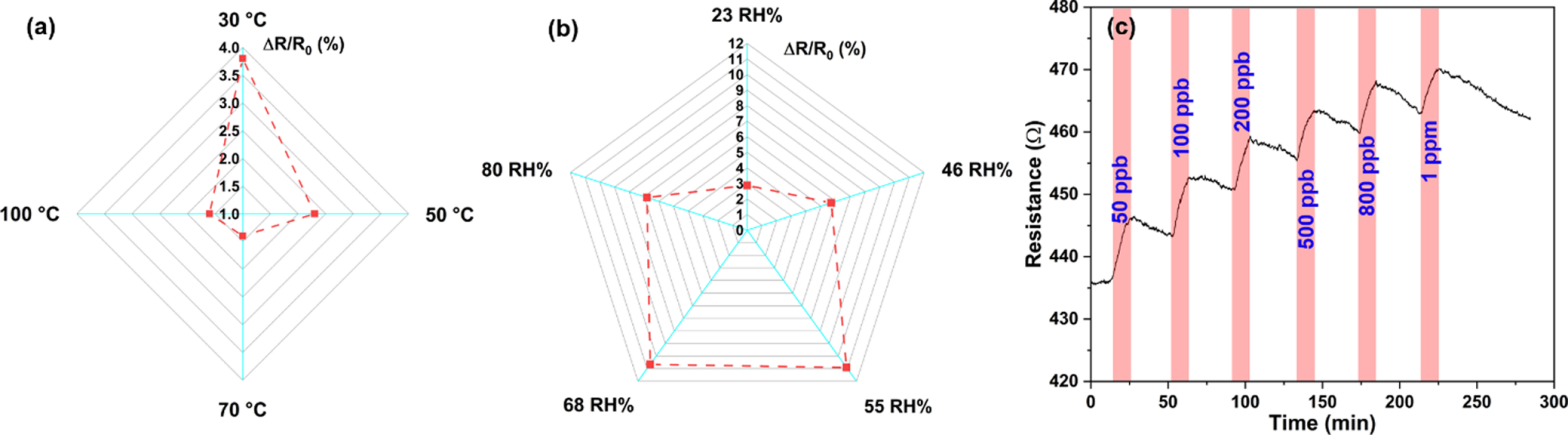
Effects of temperature (a) and humidity (b) on the sensing performance for 100 ppb H_2_S. (c) Repeated real-time sensor resistance curve for H_2_S detection after 1 month.

To study the effect of operating temperature on the sensing performance, we analyze the response for 100 ppb H_2_S at 50, 70, and 100 °C (Figure 4a). The response of the sensor is lower at higher working temperatures, which can be attributed to the reduced adsorption and enhanced desorption of the analyte at the active sites.^40,41^ On the other hand, humidity significantly increases the response of the sensor (measured in 100 ppb H_2_S) from 2.9% at 23 RH % (relative humidity) to 10.9% at 55 RH % (Figure 4b). Interestingly, the increase stops at RH 68% and is reduced at 80% RH, which may be attributed to the buildup of a physisorbed water film on the sensing composite hindering the adsorption of H_2_S molecules.^42,43^ In addition, an excess amount of surface-adsorbed water can lead to the deprotonation of H_2_S resulting in ionic moieties on the surface (H_3_O^+^, HS^–^, and S^2–^), which reduce the increase of sensor resistance.^44^

Furthermore, we assessed the stability of sensors by performing new measurements after storing the devices in a laboratory atmosphere for up to a month (Figure 4c). Upon one month of storage, the base line resistance of the device increased to about twice of its original value (Figures 4c, see S9 for the results after 1, 7, and 10 days) and it also showed a slightly higher drift during the real-time measurement. However, the magnitude of response to H_2_S pulses remains quite similar, although with the signal less dependent on the gas concentration than earlier. It is also worth noting that the color of the C_60_Br_24_/SWCNT composite transformed from yellow into brown. The change of the color and base line resistance might be caused by the presence of moisture in the air, which may promote partial decomposition of the C–Br bonds in C_60_Br_24_.^45−47^ These aging effects will be analyzed in further studies.

### 2.3 Mechanism of Sensing

**Figure 5:**
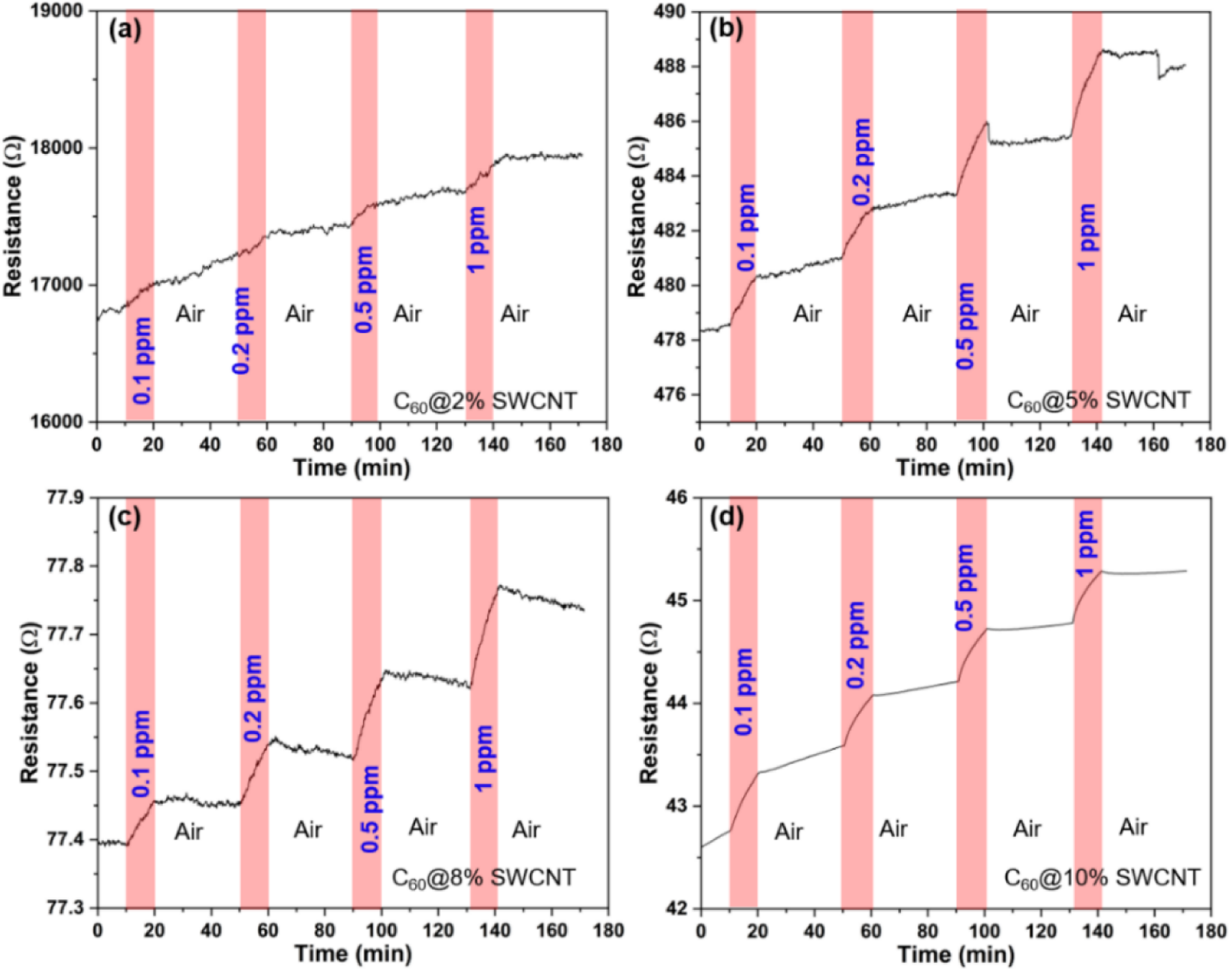
Sensing performance of composites based on pristine C_60_ with (a) 2, (b) 5, (c) 8, and (d) 10 wt % SWCNTs.

To understand the advantage of halogen bonds in the sensing process, we performed the same experiments with the C_60_/SWCNT composite (Figure 5) as with its C_60_Br_24_/SWCNT counterparts (displayed in Figure 2). While the C_60_/SWCNT composites show a clear response for H_2_S gas pulses, with the highest response of 0.90% at 0.1 ppm observed from the C_60_@10 wt % SWCNT composite, the corresponding response of the C_60_Br_24_/SWCNT composite is approximately two times higher even at a lower concentration (1.75% at 0.05 ppm). The transient response curve of the sensor based on C_60_@10 wt % SWCNTs for H_2_S sensing in the concentration range from 50 ppb to 1 ppm is shown in Figure S10. The results indicate that the presence of the C–Br and consequently the formation of a halogen bond with the analyte improve the sensing performance.

**Figure 6:**
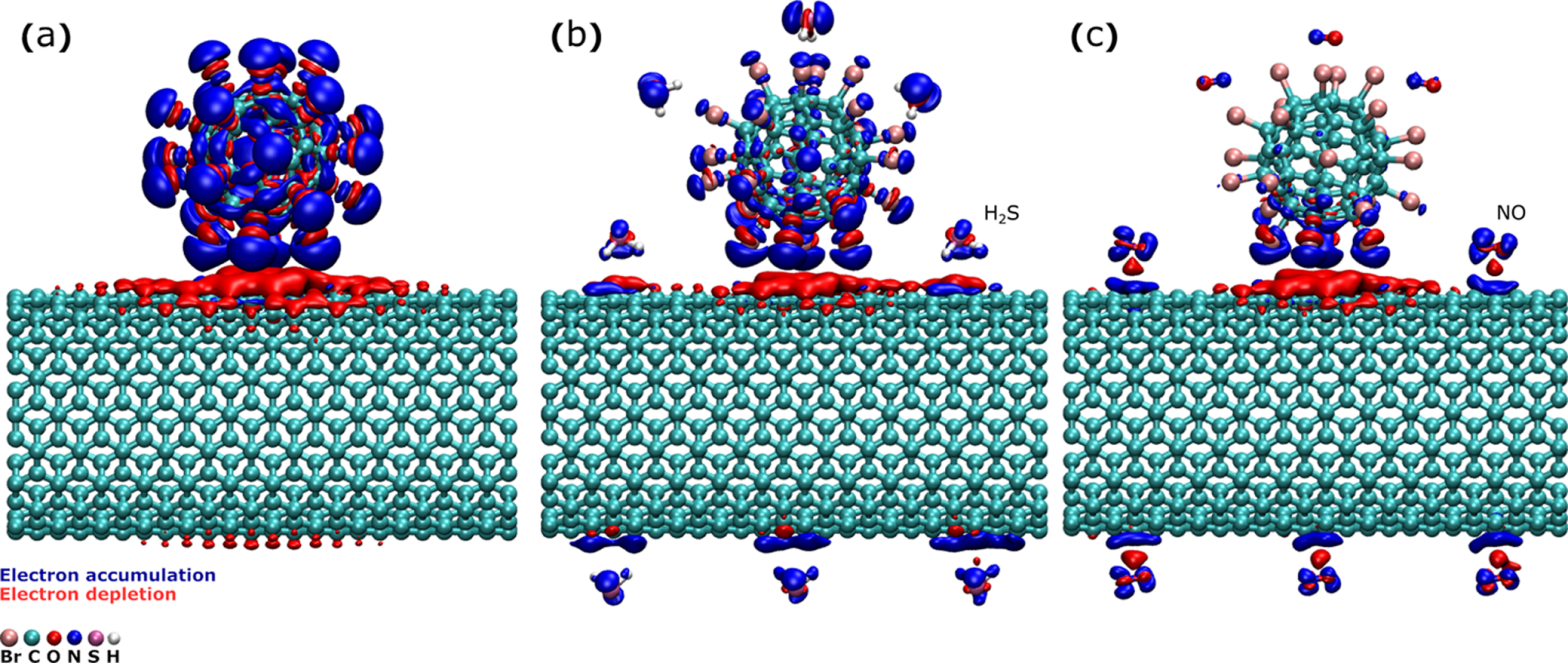
Atomic structure of the interface between C_60_Br_24_ and SWCNTs (a) without analytes, and with several adsorbed (b) H_2_S and (c) NO molecules. The charge transfer upon joining is indicated by red and blue isosurfaces, with an isovalue of 0.0002 e/bohr^3^ used in all panels.

To elaborate on the sensing mechanism, we have performed DFT calculations. In order to model the C_60_Br_24_/SWCNT interface, we placed a single C_60_Br_24_ molecule on the surface of a (10,10) SWCNT (which is metallic and has a diameter corresponding to the experimental values). The atomic structure and the resulting charge transfer upon joining the two constituents are illustrated in Figure 6a. The charge transfers and binding energies are also listed in Table 1. A remarkable transfer of 0.62 electrons from SWCNT to C_60_Br_24_ is found, owing to the lowest unoccupied molecular orbital of C_60_Br_24_ falling close to the Fermi level of the metallic SWCNTs (cf. the energy level diagram in Figure S11). Thus, SWCNTs interfaced with C_60_Br_24_ become hole-doped. Next, we considered H_2_S adsorption on SWCNTs, C_60_Br_24_, and in the vicinity of the C_60_Br_24_/SWCNT interface. The binding energy is moderately large at the SWCNTs and near the C_60_Br_24_/SWCNT interface, suggesting that those are the dominant adsorption sites (cf. adsorption geometries in Figure S12). When H_2_S adsorbs on the nanotubes, it acts as an electron donor leading to local electron doping (Figure 6b). Such a spatial modulation of the hole concentration along the SWCNTs results in potential barriers (and scattering centers) in the longitudinal direction, which inherently causes a reduced conductivity of the SWCNTs and also their percolated network.

**Table 1:** Charge Transfer and Binding Energy Upon Joining CNTs and C_60_Br_24_ or Upon Adsorption of Gas Molecules^a^

structure (A@B)	charge transfer (*e*)	binding energy (eV)
C_60_Br_24_@SWCNT	0.616	–1.126
SWCNT@H_2_S	0.011	–0.274
C_60_Br_24_@H_2_S	0.037	–0.014
SWCNT/C_60_Br_24_@H_2_S	0.004 (0.642)	–0.352
C_60_@H_2_S	0.004	–0.001
SWCNT@NO	0.014	–0.061
C_60_Br_24_@NO	0.137	–0.113
SWCNT/C_60_Br_24_@NO	0.066 (0.831)	–0.524
C_60_@NO	0.068	–0.030

a For structure A@B, charge transfer is defined as electrons transferred from B to A. The value in parentheses refers to charge transfer in C_60_Br_24_@CNT in the presence of H_2_S or NO. Binding energy is defined as *E*_B_ = *E*_Sens+Mol_ – (*E*_Sens_ + *E*_Mol_), where *E*_Sens+Mol_ is the total energy of the sensor with the adsorbed molecule, *E*_Sens_ is the energy of the sensor, and *E*_Mol_ is the energy of molecules.

It is worth noting that in our experiments, exposure of the sensor to NO molecules (known to have a typically oxidizing character) has shown an increase of the resistance (Figure 3a), which is counterintuitive, as one may expect to see its opposite effect. Therefore, we performed calculations also for the interaction of NO with the sensing material to understand the underlying causes. Interestingly, the simulations indicate that also NO behaves as a weak electron donor (Figure 6c, Table 1 and Figure S13). The binding energy was the largest at the interface, highlighting the importance of the C_60_Br_24_/SWCNT interface for sensing. In the end, conductivity is expected to change in the same direction as for H_2_S, in agreement with our experimental observations.

While semiconducting tubes are also present in the sensor composites, only metallic nanotubes were considered in the calculations. Due to the very good percolation of nanotubes in the composite and very low resistance of the sensors (Figure 2b–d), most of the current in such sensors is expected to flow across the metallic nanotubes and little (if any) through the semiconducting ones. Moreover, the *I*–*V* curves in Figure S2 show Ohmic contacts, which also supports that the conduction occurs through metallic CNTs and junctions.

Furthermore, although we have not performed calculations for semiconducting CNTs (known to exhibit a p-type character), intuitively the mechanism of sensing shall be very similar to the one described above. Because the presence of a band gap is known to result in reduced conductivity, the overall transport is expected to be even more sensitive to inhomogeneous electron and hole doping by the analyte than that in the metallic nanotubes.

Furthermore, bromination of SWCNTs alone (i.e., without applying C_60_Br_24_) cannot effectively improve the sensing performance of SWCNTs (Figure S14) for H_2_S detection because the structure lacks spatial modulation of electron-rich and depleted regions along the nanotubes necessary for sensing as described by our model.

## 3 Conclusions

In our study, we reported a new type of sensing medium to detect gaseous analytes at room temperature. C_60_Br_24_ synthesized by the bromination of C_60_ in the presence of a FeBr_3_ catalyst was mixed with (SWCNTs) to achieve conductive composites. Because of Br in C_60_Br_24_, the material might be capable of forming halogen bonds of medium strength with H atoms of various gas molecules thereby yielding sensors with good gas adsorption, a high response, and reasonable recovery properties at room temperature. As we found, non-halogenated fullerenes mixed with SWCNTs were poor sensing materials, whereas devices based on the composites of C_60_Br_24_ and SWCNTs exhibited excellent sensing properties. In particular, high selectivity with excellent response (1.75% at 50 ppb) and a low experimental detection limit (25 ppb) for H_2_S was measured in contrast to other analytes (H_2_, CO, CH_4_, NO, and NH_3_). According to DFT simulations, the carrier transport in the CNTs is influenced by the modulation of carrier density along the nanotubes. Namely, the CNTs at the C_60_Br_24_ contact are p-doped, whereas locations at which H_2_S adsorbs directly on the CNTs are less positive due to the electron doping from H_2_S. Therefore, the spatial fluctuation of the hole concentration along the CNTs is amplified upon gas adsorption, which results in the formation of potential barriers (and scattering centers) in the longitudinal direction leading to reduced conductivity of the CNTs together with their percolated network.

## 4 Experimental Section

### 4.1 Materials and Characterization

SWCNTs (diameter: 1.2–1.5 nm, length: 2–5 μm, and *I*_D_/*I*_G_ = 0.02), fullerene, Br_2_ liquid, FeBr_3_, and ethanol were purchased from Sigma-Aldrich. XRD measurements were carried out using a Multiple Crystals X-ray Diffractometer (XRD, Siemens D5000, Cu Kα radiation) with a step of 0.02°. XPS measurements were performed with a Kratos Axis Supra equipped with an Al Kα source. The microstructure of the synthesized material was studied using field-emission scanning electron microscopy (FESEM, Zeiss ULTRA plus). The TGA (Setaram Labsys) was conducted with air as the carrier gas at a heating rate of 10 °C/min from room temperature to 800 °C. The Raman spectra were recorded using a confocal microscope (Bruker Sentera II, λ = 532 nm excitation). The infrared spectra were collected using a Bruker Vertex 70 FT-IR unit in transmission mode with pure KBr as the background.

### 4.2 Synthesis of C_60_Br_24_ and Br-SWCNT

The procedure similar to that described by Djordjević and co-workers was adopted.^32^ In short, 300 mg (0.42 mmol) fullerene and 2 mL bromine liquid were stirred in the presence of 10 mg FeBr_3_ for 2 h at room temperature. After completing the reaction, the mixture was poured into a mixture of ethanol and H_2_O (1:2), vacuum filtered, and washed with the same mixture of ethanol and H_2_O. The obtained C_60_Br_24_ was dried in an oven at 80 °C for 24 h. Br-SWCNT was synthesized with the same method as that used for C_60_Br_24_. To prove that Br-SWCNT was synthesized successfully, FTIR and Raman spectra were collected (Figure S15).

### 4.3 Preparation of C_60_Br_24_/SWCNT Composites

SWCNTs and C_60_Br_24_ with different mass ratios of 2, 5, 8 and 10 wt % were dispersed in 5 mL chloroform separately using ultrasonication for 30 min, and the composite was collected by vacuum filtration.

### 4.4 Sensor Preparation

The process for fabricating sensors is as follows: 20 mg of composite was mixed with 0.5 mL of chloroform to form a paste, which was then brush-coated onto an Al_2_O_3_ substrate printed with five pairs of Ag–Pd interdigitated electrodes (IDES, electrode distance and width were both 200 μm) (14 mm × 7 mm) to form a sensitive film and dried at 70 °C for 20 min.

### 4.5 Sensing Measurements

The performance of the sensor based on C_60_Br_24_/SWCNT was characterized by measuring the resistance of the sensor exposed to different concentrations of the gas. The changes in the resistance were monitored through a Linkam THMS600 heating and freezing stage connected to an Agilent 3458A multimeter at a constant bias of 5 V, and different concentrations of H_2_S, NH_3_, NO, CH_4_, CO, and H_2_ were prepared using a dilution system controlled by a computer. Commercial dry synthetic air was used as the carrier to dilute these gases to the desired concentrations, and room temperature (∼25 °C) was maintained to be the operating temperature in this work. Specifically, the sensor was placed on the heating and freezing stage in a sealed chamber with an electric feed-through between a gas inlet and a gas outlet. To carry out measurements and compare the response in a convenient way, the exposure time was chosen as 10 min and the purging time was fixed at 30 min per pulse. To exclude the interference on the resistance from the different gas flow rates, in this work, the total flow of gas into the chamber was controlled at a rate of 500 mL/min. To add water vapor to the test gas, an additional air flow was bubbled through a water-containing flask and mixed to the analyte stream before inserting to the test chamber. The relative humidity was controlled by adjusting the carrier gas flow rates. The final humidity of the test gas was calibrated using a commercial humidity sensor.

### 4.6 DFT Calculations

All the calculations are carried out using DFT as implemented in the CP2K software.^48−51^ The PBE functional^52^ is used with Goedecker–Teter–Hutter (GTH) pseudopotentials^53,54^ and Gaussian-type MOLOPT basis sets (DZVP-MOLOPT-GTH).^55^ The D3 correction is used to describe vdW interactions^56^ and a cutoff energy of 800 Ry is set for the plane-wave expansion of the electron density. Comparison of the calculated bond lengths in C_60_ and C_60_Br_24_ to those reported in the literature is listed in Tables S1 and S2. The threshold for energy convergence is set to 10^–12^ Ha and the one related to SCF cycles is set to 10^–6^ Ha. A Fermi–Dirac smearing with an electronic temperature of 2000 K of the occupation number at the Fermi level is used except for NO that was 0 K. For SWCNTs, we use (10,10) chirality and a 12× supercell in the longitudinal direction. The Brillouin zone is sampled using the sole Γ-point. Charge transfer is determined using Mulliken population analysis.^57^ For finding the structure of both H_2_S and NO on both SWCNTs and C_60_Br_24_, we selected nine initial configurations with different positions and molecule orientations. In the case of the SWCNT/C_60_Br_24_ model, we considered the same nine configurations and two additional (11 total). The atom positions were then optimized, but not lattice constants. The bond lengths for the lowest energy structures were then compared to previous computational and experimental results, where available, and were found to agree with them. Binding energy and charge transfer for three other configurations of H_2_S and NO adsorbed on SWCNT/C_60_Br_24_ are listed in Table S3 and the atomic structures are shown in Figures S12 and S13.

## Supporting Information Available

XRD, TGA, repeatability, and sensing performance of pure SWCNTs and C_60_/SWCNTs; two definitions of response applied in the article; the measurements of noise and the calculation method of LOD; simulation information including the energy level diagram of each material; and adsorption model of H_2_S and NO and bond length in C_60_Br_24_ (PDF)
