# Supplementary material for: C60Br24/SWCNT: A Highly Sensitive Medium to Detect H2S via Inhomogeneous Carrier Doping
Source: ACS Appl Mater Interfaces. 2021 Dec 6;13(49):59067–75. doi: 10.1021/acsami.1c16807 (PMC8678982; doi:10.1021/acsami.1c16807)
Supplement: Supplementary file 1 — am1c16807_si_001.pdf [file am1c16807_si_001.pdf]

Supporting Information

$C_{60}Br_{24}$/SWCNT: A highly sensitive medium to detect H_2S via inhomogeneous carrier doping

Jin Zhou, Mohammad Bagheri, Topias Järvinen, Cora Pravda Bartus, Akos Kukovecz, Hannu-Pekka Komsa and Krisztian Kordas**

J. Zhou, M. Bagheri, T. Järvinen, H.-P. Komsa, K. Kordas
Microelectronics Research Unit, Faculty of Information Technology and Electrical Engineering, University of Oulu, P.O. Box 4500, FIN-90014 Oulu, Finland
E-mails: Krisztian.Kordas@oulu.fi and Hannu-Pekka.Komsa@oulu.fi

C.P. Bartus, A. Kukovecz
Interdisciplinary Excellence Centre, Department of Applied and Environmental Chemistry, University of Szeged, Rerrich Béla tér 1, H-6720 Szeged, Hungary

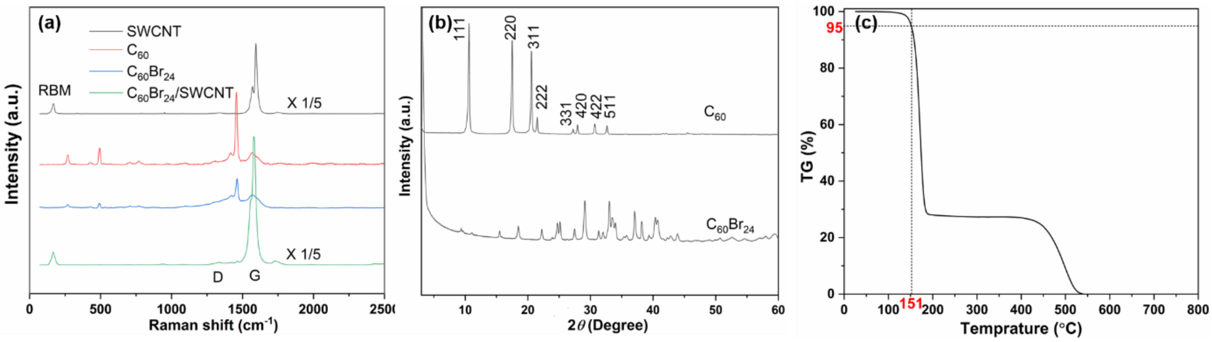

Figure S1. (a) Raman spectra of the SWCNTs, C_{60}, $C_{60}Br_{24}$, and $C_{60}Br_{24}$/SWCNT composite; (b) XRD patterns of C_{60} and $C_{60}Br_{24}$, and (c) TGA curve of $C_{60}Br_{24}$.

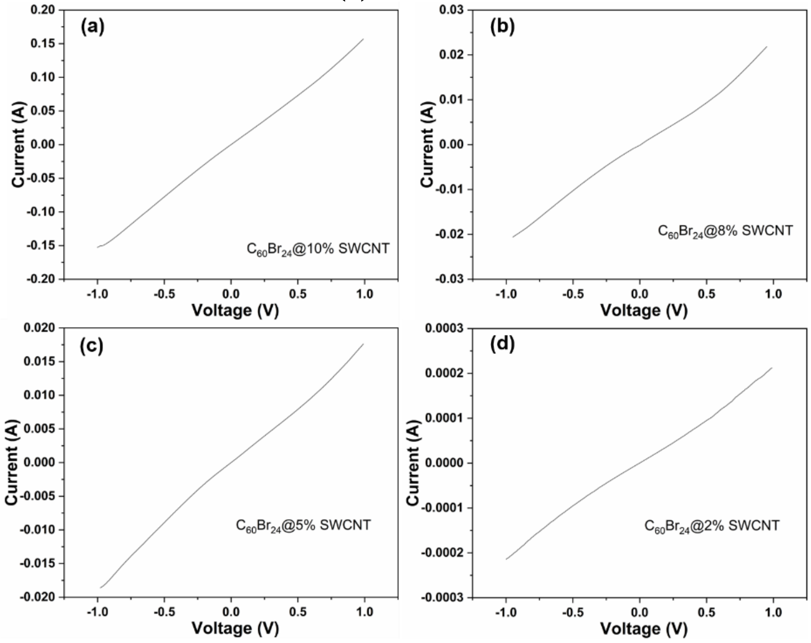

Figure S2. I-V characteristics of devices based on the (a) $C_{60}Br_{24}$@10% SWCNT, (b) $C_{60}Br_{24}$@8% SWCNT, (c) $C_{60}Br_{24}$@5% SWCNT and (d) $C_{60}Br_{24}$@2% SWCNT

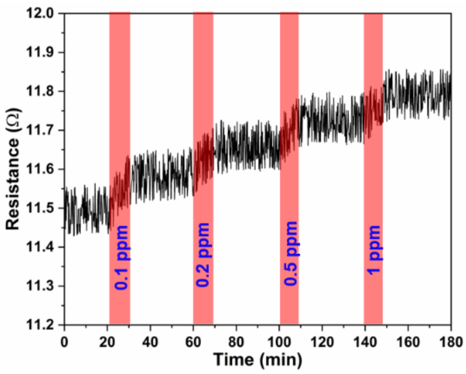

Figure S3. H_2S sensing performance of a device based on pristine SWCNTs as active sensing layer.

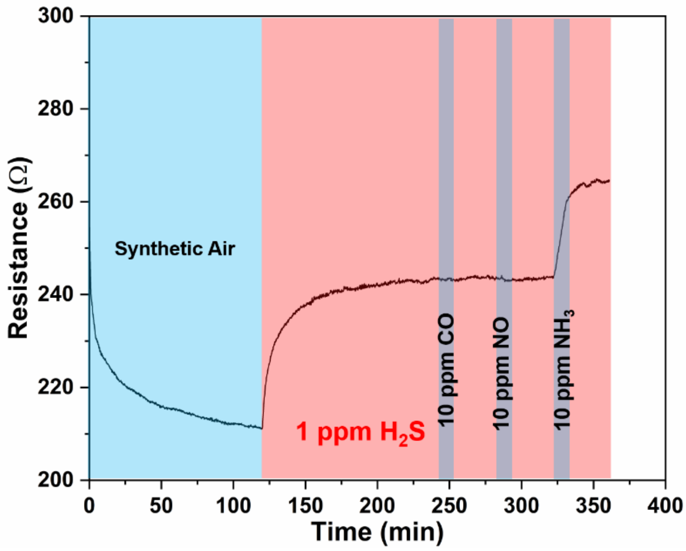

Figure S4. Cross-selectivity of the sensor device. The sensor is constantly exposed to 1 ppm H_2S from 120 min to the end.

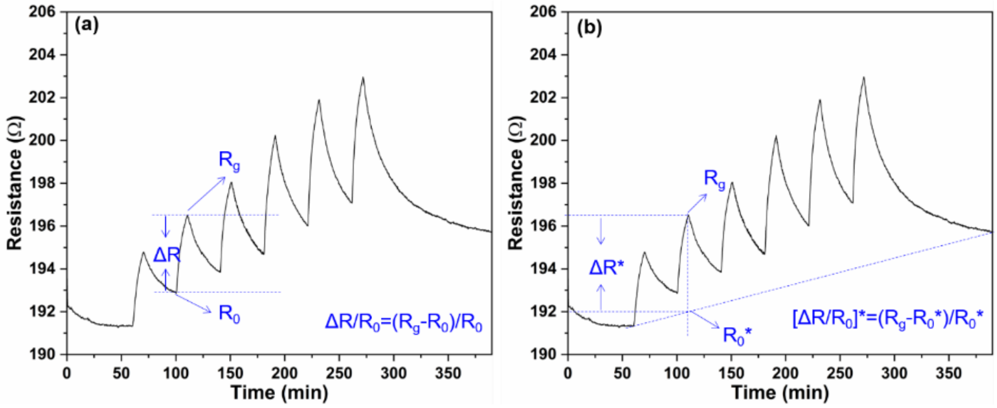

Figure S5. Calculation methods applied for determining the sensor response curves in Figure 3. Method (a) neglects baseline drift thus overestimates the response value. On the other hand, method (b) underestimates the response because of the imperfect sensor recovery between subsequent gas pulses.

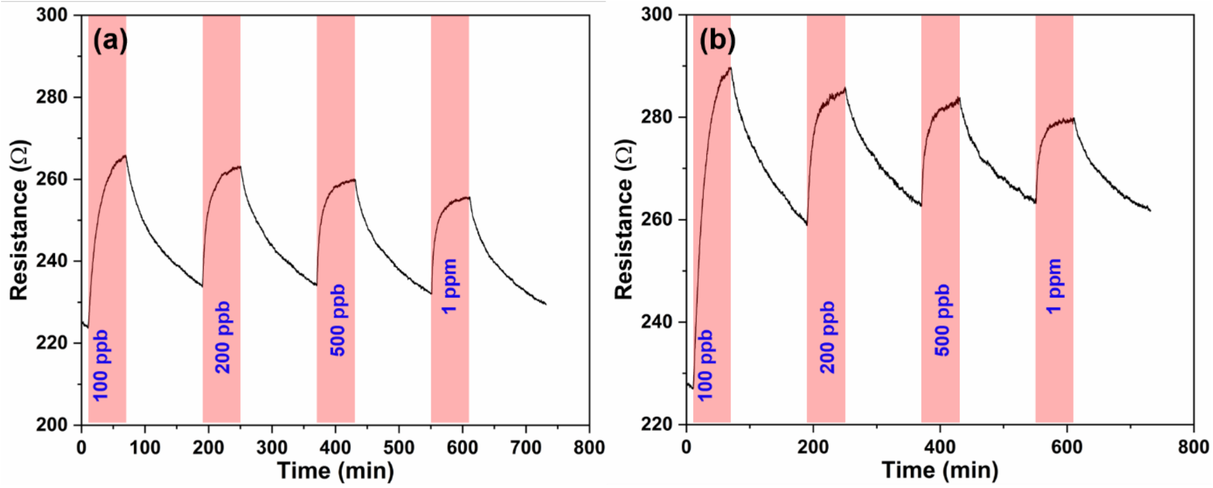

Figure S6. Sensing performance of sensor for 1 h exposure and 2 h recovery at different H_2S concentrations. Curve (b) was measured two days after curve (a) using the same device.

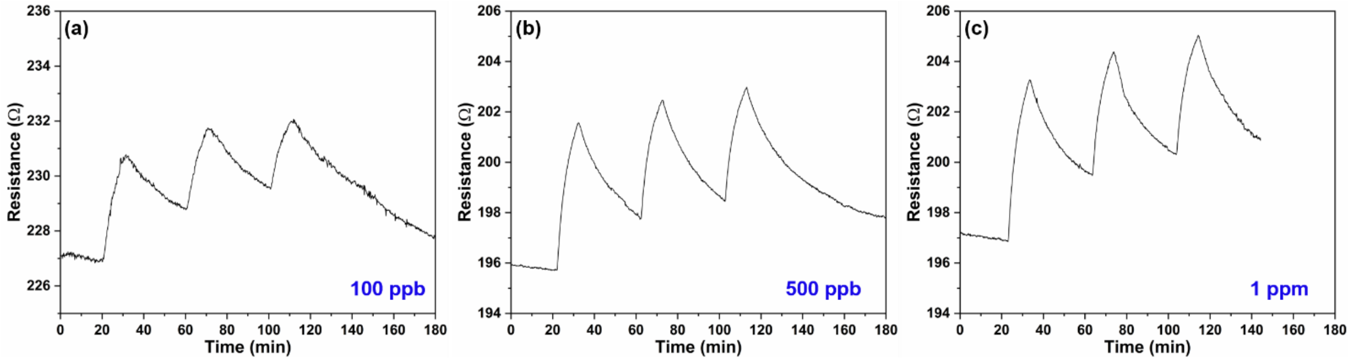

Figure S7. Repeatability of sensor response based on the $C_{60}Br_{24}$/SWCNT composite at (a) 100 ppb, (b) 500 ppb and (c) 1 ppm H_2S.

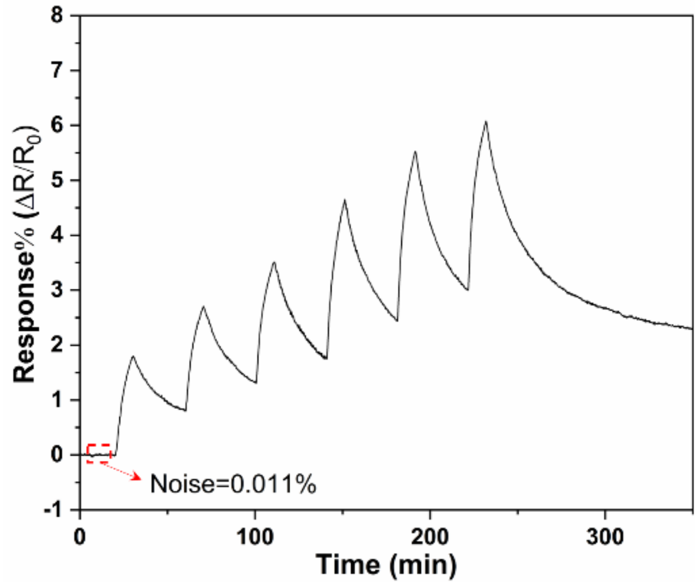

Figure S8. Noise occurred in the process of sensing measurement. The noise is defined as the standard deviation of the datapoint without the gas analytes exposure. The LOD for H_2S was calculated as following: LOD=3×SD/sensitivity=3×0.011%/(35% ppm^{-1}) ≈1 ppb.

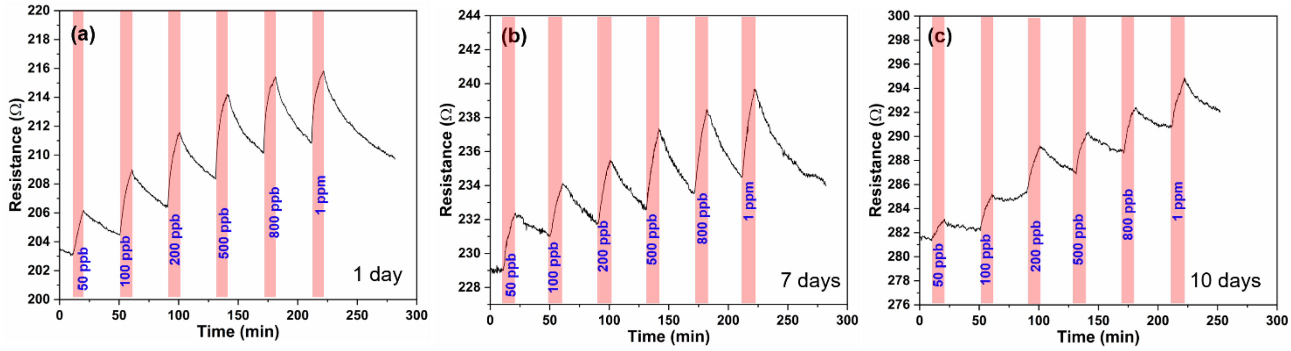

Figure S9. Sensing performance of the sensor device after (a) 1 day, (b) 7 days, and (c)10 days of ageing.

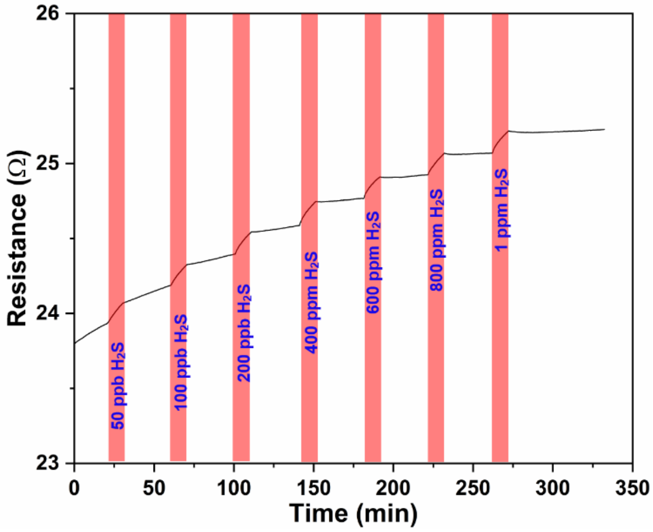

Figure S10. Real-time sensor resistance curve of a device with C_{60}@10%SWCNT composite.

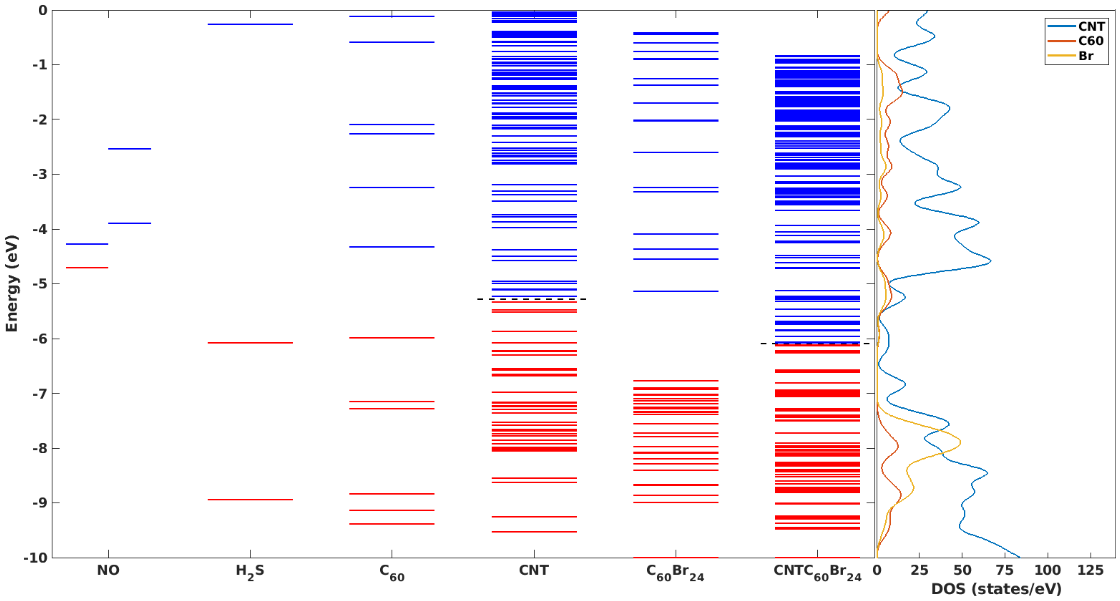

Figure S11. Energy level diagram of each modeled structure. The red and blue lines correspond to occupied and unoccupied states, respectively, and the black dashed lines illustrate the Fermi level position in the (semi-)metallic systems. In the case of NO, the two sets of lines correspond to spin-up and spin-down components. Panel on the right shows the partial density of states of SWCNT/$C_{60}Br_{24}$, to give an idea which states belong to SWCNT and which to $C_{60}Br_{24}$. The Fermi-level remains close to the Dirac-point of SWCNT but close to the LUMO of $C_{60}Br_{24}$.

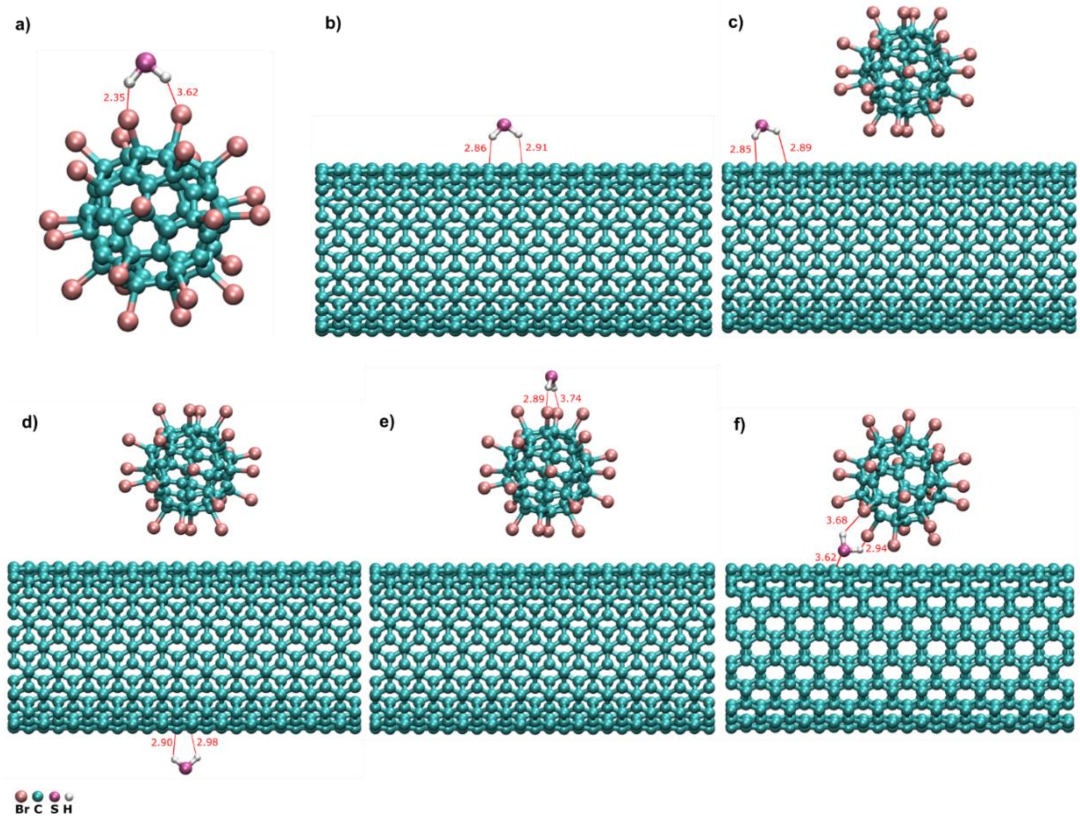

Figure S12. Atomic structures of H_2S adsorbed on (a) $C_{60}Br_{24}$, (b) SWCNT, (c) side of SWCNT/$C_{60}Br_{24}$ interface, (d) bottom of SWCNT/$C_{60}Br_{24}$, (e) top of SWCNT/$C_{60}Br_{24}$, and (f) at the SWCNT/$C_{60}Br_{24}$ interface. Selected bond lengths are also indicated.

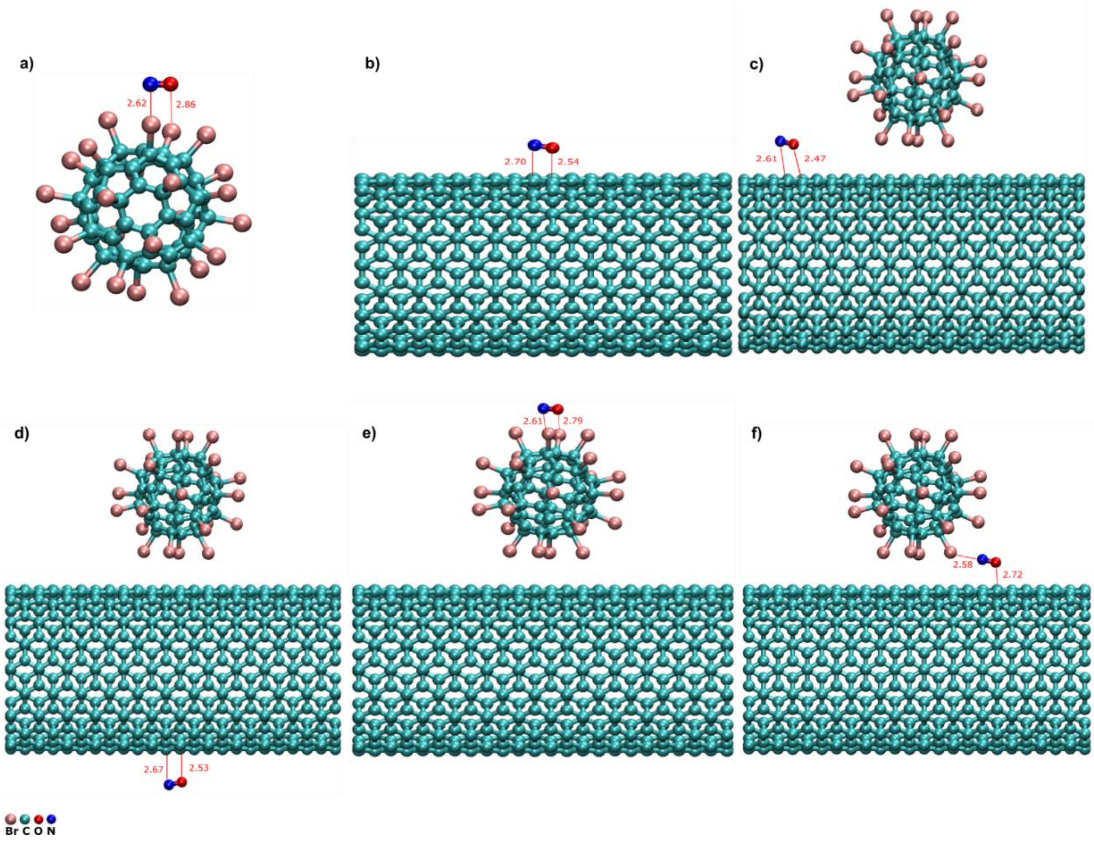

Figure S13. Atomic structures of NO adsorbed on (a) $C_{60}Br_{24}$, (b) SWCNT, (c) side of SWCNT/$C_{60}Br_{24}$ interface, (d) bottom of SWCNT/$C_{60}Br_{24}$, (e) top of SWCNT/$C_{60}Br_{24}$, and (f) at the SWCNT/$C_{60}Br_{24}$ interface. Selected bond lengths are also indicated.

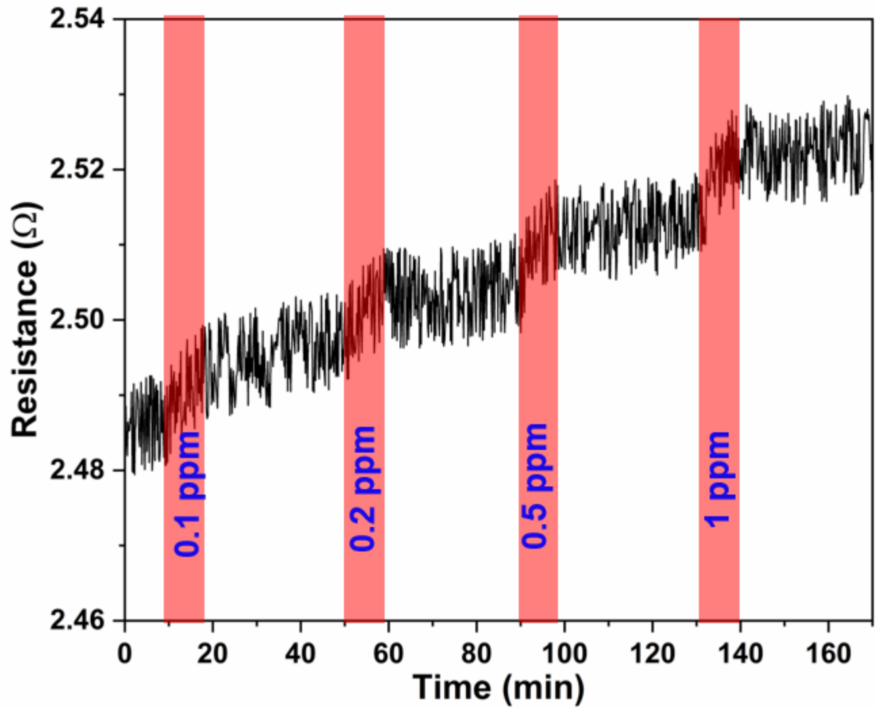

Figure S14. Real-time sensor resistance curve of a device with Br-SWCNT as active layer.

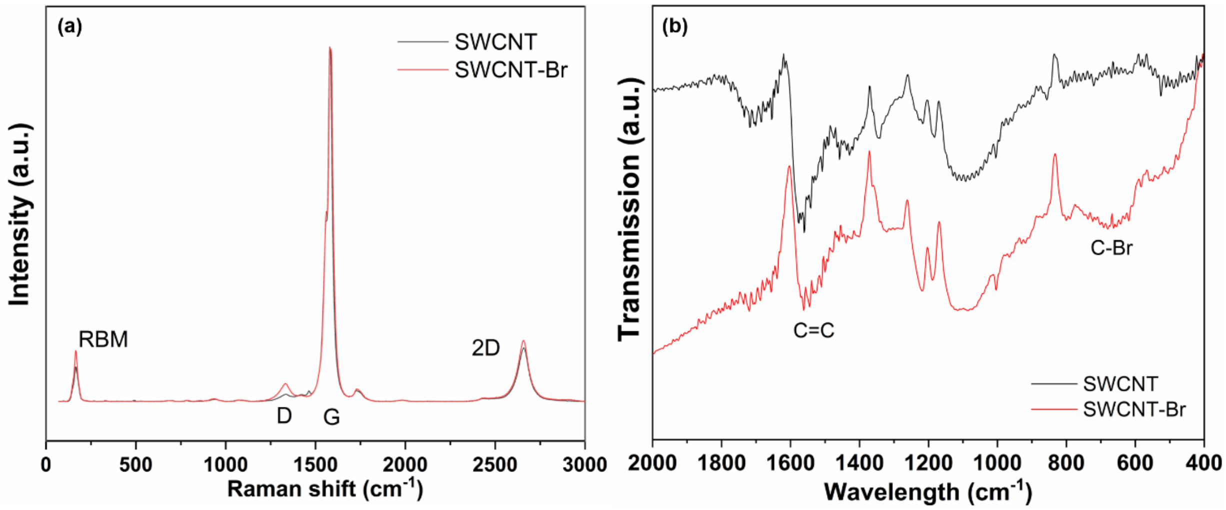

Figure S15. (a) Raman and (b) FTIR spectra of brominated SWCNT (SWCNT-Br) and pristine SWCNT. D-band of SWCNT-Br exhibits broadening and upshifting compared to pure SWCNT, which arised from charge transfer and the formation of vacancies after brimnation, respectivley. C-Br peak at 670 cm^{-1} can be found in FTIR of SWCNT-Br, indicating the successful brimination of SWCNT.[1]

Table S1. Bond lengths of C_{60} calculated with different exchange-correlation functionals and comparison to the calculated and experimental values reported in the literature.

	C=C	C-C	ref
ab initio	1.37	1.45	2
Expt	1.39	1.44	3
LDA	1.387	1.439	computed
PBE	1.399	1.451	computed
PBE-D3	1.384	1.436	computed

Table S2. Bond lengths of $C_{60}Br_{24}$ calculated with different exchange-correlation functionals and comparison to the calculated and experimental values reported in the literature.

Basis set	C=C (a)	C=C (b)	C-C (c)	C-C (d)	C-C (e)	C-Br	Ref
6-31G	1.316	1.325	1.486	1.506	1.505	1.992	4
(6-31G, SBKJC)	1.318	1.329	1.492	1.512	1.511	2.032	4
6-31G*	1.314	1.32	1.497	1.511	1.511	1.961	4
(6-31G*, SBKJC*)	1.314	1.323	1.5	1.514	1.512	1.979	4
Experimental X-ray b [1]	1.338	1.339	1.489	1.512	1.5	1.993	5
Experimental X-ray b [2]	1.338	1.35	1.503	1.511	1.516	1.994	6
LDA	1.333	1.344	1.468	1.488	1.492	2.018	computed
PBE	1.344	1.357	1.486	1.505	1.508	2.061	computed
PBE-D3	1.328	1.340	1.467	1.487	1.490	2.017	computed

Table S3. Charge transfer and binding energy upon joining CNT and $C_{60}Br_{24}$ or upon adsorption of gas molecules in several configurations. The first three values are the same as those listed in Table 1 of the main paper. The charge transfer and binding energy are defined as in Table 1.

Structure (A@B)	Charge transfer (e)	Binding energy (eV)
H_2S		
SWCNT@H_2S	0.011	-0.274
$C_{60}Br_{24}$@H_2S	0.037	-0.014
SWCNT/$C_{60}Br_{24}$@H_2S (interface)	0.004	-0.352
SWCNT/$C_{60}Br_{24}$@H_2S (bottom of SWCNT)	0.009	-0.172
SWCNT/$C_{60}Br_{24}$@H_2S (top of $C_{60}Br_{24}$)	0.042	-0.017
SWCNT/$C_{60}Br_{24}$@H_2S (side of interface)	0.014	-0.165
NO		
SWCNT@NO	0.014	-0.061
$C_{60}Br_{24}$@NO	0.137	-0.113
SWCNT/$C_{60}Br_{24}$@NO (interface)	0.066	-0.524
SWCNT/$C_{60}Br_{24}$@NO (bottom of SWCNT)	0.01	-0.057
SWCNT/$C_{60}Br_{24}$@NO (top of $C_{60}Br_{24}$)	0.157	-0.258
SWCNT/$C_{60}Br_{24}$@NO (side of interface)	0.084	-0.157

References

(1) Hines, D.; Rümmeli, M. H.; Adebimpe, D.; Akins, D. L. High-Yield Photolytic Generation of Brominated Single-Walled Carbon Nanotubes and Their Application for Gas Sensing. *Chem. Commun.* **2014**, *50* (78), 11568–11571.

(2) Kvyatkovskii, O. E.; Shelyapina, M. G.; Shchegolev, B. F.; Vorotilova, L. S.; Zakharova, I. B. Cluster Ab Initio Calculations for the $C_{60}F_{24}$, $C_{60}Cl_{24}$, and $C_{60}Br_{24}$ Halofullerenes. *Phys. Solid State* **2002**, *44* (3), 585–587.

(3) Eletskii, A. V; Smirnov, B. M. Fullerenes and Carbon Structures. *Physics-Uspekhi* **1995**, *38* (9), 935.

(4) Popov, A. A.; Senyavin, V. M.; Granovsky, A. A. Vibrations of Bromofullerene $C_{60}Br_{24}$: C_{60} Cage Confined into Static Bromine Sphere. *Chem. Phys. Lett.* **2004**, *383* (1–2), 149–155.

(5) Tebbe, F. N.; Harlow, R. L.; Chase, D. B.; Thorn, D. L.; Campbell, G. C.; Calabrese, J. C.; Herron, N.; Young, R. J.; Wasserman, E. Synthesis and Single-Crystal X-Ray Structure of a Highly Symmetrical C_{60} Derivative, $C_{60}Br_{24}$. *Science (80-.).* **1992**, *256* (5058), 822–825.
(6) Troyanov, S. I.; Troshin, P. A.; Boltalina, O. V; Kemnitz, E. Bromination of [60] Fullerene. II. Crystal and Molecular Structure of [60] Fullerene Bromides, $C_{60}Br_6$, $C_{60}Br_8$, and $C_{60}Br_{24}$. *Fullerenes, Nanotub. Carbon Nanostructures* **2003**, *11* (1), 61–77.
